# Supplementary material for: Cosmic-ray bath in a past supernova gives birth to Earth-like planets
Source: Sci Adv. 2025 Dec 10;11(50):eadx7892. doi: 10.1126/sciadv.adx7892 (PMC12694037; doi:10.1126/sciadv.adx7892)
Supplement: Supplementary file 1 — Supplementary Text References [file sciadv.adx7892_sm.pdf]

Science Advances
AAAS

Supplementary Materials for

Cosmic-ray bath in a past supernova gives birth to Earth-like planets

Ryo Sawada *et al.*

Corresponding author: Ryo Sawada, ryo@g.ecc.u-tokyo.ac.jp

Sci. Adv. **11**, eadx7892 (2025)
DOI: 10.1126/sciadv.adx7892

This PDF file includes:

Supplementary Text
References

Supplementary Text

Brief Summary of Previous Studies

Three main hypothetical scenarios have been proposed for the origin of SLRs in the early solar system: inheritance, irradiation, and injection.

Inheritance scenario

First, in the inheritance scenario, SLRs are already present in the protosolar molecular core before its collapse—either because they were passively mixed throughout the parent molecular cloud or because ejecta from a nearby supernova were injected into the parent molecular cloud locally. This definition thus covers both (i) passive inheritance from the natal cloud and (ii) injection of SLRs into the protosolar molecular core by a supernova shock that sweeps through the cloud after the bulk of the molecular gas has condensed.

The main drawback of this scenario is the timescale problem: the time required for the solar system formation to begin is too long. SLRs with half-lives of less than 5 million years (^{10}Be, ^{26}Al, ^{36}Cl, ^{41}Ca, ^{53}Mn, and ^{60}Fe) should decay to a negligible amount during the evolution from the molecular cloud core to the proto-Sun with the protosolar disk (*8, 9*). Ref. (*62*) recently proposed self-concentration of SLRs in a spiral-arm protostar cloud, but this still cannot deliver radionuclides with half-lives of less than 1 million years in time for CAI formation. Therefore, it seems necessary to provide SLRs in a way other than inheritance shortly before CAI formation.

Beyond timing, the physical survivability of the injection of SLRs into the protosolar molecular core event is uncertain. Supernova remnants retain shock velocities $\geq$ 100 km s^{-1} out to radii $\gtrsim$ 20 pc (*63*), and simulations show that dense cores can be dispersed by shocks stronger than $\sim$ 70 km s^{-1} (*64*). Therefore, the conditions under which SLR could be injected locally into the parent molecular cloud while avoiding destruction at the distances proposed in the meteorite literature are not self-evidently satisfied and deserve quantitative testing beyond the scope of this study.

Nevertheless, inheritance need not necessarily correspond to a single nearby event. Multiple supernovae at larger distances within the same or neighboring star-forming regions can provide a cloud-scale ^{26}Al enrichment (*65*). We should note that this pathway moderates shock-destruction of dense cores, but relies on mixing efficiencies and transport times operating on longer spatial and temporal scales. In this sense, cloud-scale inheritance may have contributed modestly to the background level of SLRs in the protosolar environment, within which our immersion mechanism, that is, shorter timescale contamination from a nearby supernova, took place.

Taken together, while cloud-scale enrichment cannot yet be ruled out, its temporal and spatial feasibility remains an open question.

Irradiation scenario

Next, the irradiation scenario proposes `in-situ' nucleosynthesis occurring in the protosolar disk due to the irradiation. ^{10}Be is one remarkable nuclide that is produced only by the energetic-particle irradiation (e.g., Ref. (*29, 61*)) and cannot be supplied by other sources. The inferred value of ^{10}Be/^{9}Be $\gtrsim 10^{-4}$ (*66, 67*) is too large to achieve by conventional Galactic Cosmic Ray irradiation (GCR-irradiation; (*29*)). Therefore, ^{10}Be in the early solar system is mainly produced by the irradiation from a proto-Sun to a protosolar disk (PS-irradiation). Charged particles accelerated from the central star, especially during solar flares, possess sufficiently high energy ($\sim$ 10-100 MeV) to cause such nucleosynthesis (*68*). Classically, the so-called X-wind model (*69*), which includes nucleosynthesis processes due to the PS-irradiation for rocky vapors, has been proposed and widely accepted. However, numerous concerns were pointed out with the X-

wind model (*70*), and it is considered that the X-wind model could not explain the solar system either from a nucleosynthesis perspective. Now Ref. (*17*) is the only viable and quantitative model, including the PS-irradiation. This model still faces a problem from the viewpoint of the homogeneity of the SLRs within the protosolar disk. Since the PS irradiation occurs in the minimal region near the central proto-Sun, it is required that an efficient transport mechanism mixes materials throughout the protosolar disk (*68*).

Injection scenario

Finally, the injection scenario suggests the possibility that the SLRs are directly injected into a protosolar system from a nearby core-collapse supernova explosion, as the lifetime of a massive star is short (less than 10 million years for stars with a mass of 20 $M_{\odot}$ or more (*71*)). The initial abundance of ^{60}Fe, which is exactly the opposite of ^{10}Be, is the key to unveiling the origin of SLRs. This is because ^{60}Fe is barely synthesized by energetic-particle irradiation around the young Sun (e.g., Ref. (*61*)). In the injection scenario, the difficulty lies in the conditions under which direct injection can be achieved without destroying the solar system. There are two possibilities: direct injection into the parental molecular cloud core (*72*) and into the protosolar disk (*28*). Ref. (*73*) argued that the chromium isotopic heterogeneity found in various chondritic meteorites might be evidence of the direct injection onto the protosolar disk. However, Ref. (*16*) ruled out direct injection into the protosolar disk as the only explanation for the origin of SLRs, such as an insufficient amount of ^{26}Al.

REFERENCES AND NOTES

1. T. Lichtenberg, J. Drazkowska, M. Schonbachler, G. J. Golabek, T. O. Hands, Bifurcation of planetary building blocks during Solar System formation. *Science* **371**, 365–370 (2021).

2. M. Newcombe, S. Nielsen, L. Peterson, J. Wang, C. O. Alexander, A. Sarafian, K. Shimizu, L. Nittler, A. Irving, Degassing of early-formed planetesimals restricted water delivery to Earth. *Nature* **615**, 854–857 (2023).

3. H. C. Urey, The cosmic abundances of potassium, uranium, and thorium and the heat balances of the Earth, the Moon, and Mars. *Proc. Natl. Acad. Sci. U.S.A.* **41**, 127–144 (1955).

4. D. S. Grewal, N. X. Nie, B. Zhang, A. Izidoro, P. D. Asimow, Accretion of the earliest inner Solar System planetesimals beyond the water snowline. *Nat. Astron.* **8**, 290–297 (2024).

5. S. J. Desch, E. D. Young, E. T. Dunham, Y. Fujimoto, D. R. Dunlap, "Short-lived radionuclides in meteorites and the Sun's birth environment," in *Protostars and Planets VII* (Astronomical Society of the Pacific, 2023), pp. 759–798.

6. T. Lichtenberg, G. J. Golabek, R. Burn, M. R. Meyer, Y. Alibert, T. V. Gerya, C. Mordasini, A water budget dichotomy of rocky protoplanets from ^{26}Al-heating. *Nat. Astron.* **3**, 307–313 (2019).

7. T. Lee, D. A. Papanastassiou, G. J. Wasserburg, Demonstration of ^{26}Mg excess in Allende and evidence for ^{26}Al. *Geophys. Res. Lett.* **3**, 41–44 (1976).

8. S. B. Jacobsen, "The birth of the Solar System in a molecular cloud: Evidence from the isotopic pattern of short-lived nuclides in the early Solar System," in *Chondrites and the Protoplanetary Disk* (Astronomical Society of the Pacific, 2005), pp. 548–557.

9. G. R. Huss, B. S. Meyer, G. Srinivasan, J. N. Goswami, S. Sahijpal, Stellar sources of the short-lived radionuclides in the early solar system. *Geochim. Cosmochim. Acta.* **73**, 4922–4945 (2009).

10. E. D. Young, Inheritance of solar short- and long-lived radionuclides from molecular clouds and the unexceptional nature of the solar system. *Earth Planet. Sci. Lett.* **392**, 16–27 (2014).

11. S. Sahijpal, J. N. Goswami, Refractory phases in primitive meteorites devoid of ^{26}Al and ^{41}Ca: Representative samples of first solar system solids? *Astrophys J.* **509**, L137–L140 (1998).

12. A. G. W. Cameron, J. W. Truran, The supernova trigger for formation of the solar system. *Icarus* **30**, 447–461 (1977).

13. J. C. Forbes, J. Alves, D. N. C. Lin, A Solar System formation analogue in the Ophiuchus star-forming complex. *Nat. Astron.* **5**, 1009–1016 (2021).

14. N. Ouellette, S. J. Desch, J. J. Hester, L. A. Leshin, "A nearby supernova injected short-lived radionuclides into our protoplanetary disk," in *Chondrites and the Protoplanetary Disk* (Astronomical Society of the Pacific, 2005), pp. 527–538.

15. G. J. Wasserburg, M. Busso, R. Gallino, K. M. Nollett, Short-lived nuclei in the early Solar System: Possible AGB sources. *Nucl. Phys. A* **777**, 5–69 (2006).

16. R. Sawada, T. Taki, H. Kurokawa, Y. Suwa, Self-consistent conditions for ^{26}Al injection into a protosolar disk from a nearby supernova. *Astrophys J.* **963**, 68 (2024).

17. E. Jacquet, Beryllium-10 production in gaseous protoplanetary disks and implications for the astrophysical setting of refractory inclusions. *Astron. Astrophys.* **624**, A131 (2019).

18. A. M. Davis, K. D. McKeegan, "Short-lived radionuclides and early solar system chronology" in *Meteorites and Cosmochemical Processes*, A. M. Davis, Ed. (Elseiver, 2014), vol. **1**, pp. 361–395.

19. M. Lugaro, U. Ott, ´A. Kereszturi, Radioactive nuclei from cosmochronology to habitability. *Prog. Part. Nucl. Phys.* **102**, 1–47 (2018).

20. E. Fermi, On the origin of the cosmic radiation. *Phys. Rev.* **75**, 1169–1174 (1949).

21. A. R. Bell, The acceleration of cosmic rays in shock fronts I. *Mot. Not. R. Astron. Soc.* **182**, 147–156 (1978).

22. L. O. Drury, An introduction to the theory of diffusive shock acceleration of energetic particles in tenuous plasmas. *Rep. Prog. Phys.* **46**, 973–1027 (1983).

23. Y. Ohira, K. Murase, R. Yamazaki, Escape-limited model of cosmic-ray acceleration revisited. *Astron. Astrophys.* **513**, A17 (2010).

24. S. Gabici, Cosmic ray escape from supernova remnants. *Mem. Soc. Astron. Italiana* **82**, 760 (2011).

25. J. Ellis, B. D. Fields, D. N. Schramm, Geological isotope anomalies as signatures of nearby supernovae. *Astrophys J.* **470**, 1227 (1996).

26. B. D. Fields, T. Athanassiadou, S. R. Johnson, Supernova collisions with the heliosphere. *Astrophys J.* **678**, 549–562 (2008).

27. J. A. Miller, B. D. Fields, Heliospheric compression due to recent nearby supernova explosions. *Astrophys J.* **934**, 32 (2022).

28. N. Ouellette, S. J. Desch, J. J. Hester, Interaction of supernova ejecta with nearby protoplanetary disks. *Astrophys J.* **662**, 1268–1281 (2007).

29. M. Gounelle, F. H. Shu, H. Shang, A. E. Glassgold, K. E. Rehm, T. Lee, Extinct radioactivities and protosolar cosmic rays: Self-shielding and light elements. *Astrophys J.* **548**, 1051–1070 (2001).

30. G. J. MacPherson, “Calcium-aluminum-rich inclusions in chondritic meteorites” in *Meteorites and Cosmochemical Processes*, A. M. Davis, Ed. (Elseiver, 2014), vol. **1**, pp. 139–179.

31. A. J. Koning, D. Rochman, Modern nuclear data evaluation with the TALYS code system. *Nucl. Data Sheets* **113**, 2841–2934 (2012).

32. A. Koning, S. Hilaire, S. Goriely, TALYS: Modeling of nuclear reactions. *Eur. Phys. J. A* **59**, 131 (2023).

33. S. Gabici, C. Evoli, D. Gaggero, P. Lipari, P. Mertsch, E. Orlando, A. Strong, A. Vittino, The origin of Galactic cosmic rays: Challenges to the standard paradigm. *Int. J. Mod. Phys. D* **28**, 1930022–339 (2019).

34. K. Murase, M. Fukugita, Energetics of high-energy cosmic radiations. *Phys. Rev. D* **99**, 063012 (2019).

35. N. T. Kita, Q.-Z. Yin, G. J. MacPherson, T. Ushikubo, B. Jacobsen, K. Nagashima, E. Kurahashi, A. N. Krot, S. B. Jacobsen, ^{26}Al-^{26}Mg isotope systematics of the first solids in the early solar system. *Meteorit. Planet. Sci.* **48**, 1383–1400 (2013).

36. C. Hayashi, Structure of the solar nebula, growth and decay of magnetic fields and effects of magnetic and turbulent viscosities on the nebula. *Prog. Theor. Phys. Suppl.* **70**, 35–53 (1981).

37. M. Padovani, D. Galli, A. E. Glassgold, Cosmic-ray ionization of molecular clouds. *Astron. Astrophys.* **501**, 619–631 (2009).

38. F. C. Adams, The birth environment of the Solar System. *Annu. Rev. Astron. Astrophys.* **48**, 47–85 (2010).

39. S. Arakawa, E. Kokubo, Number of stars in the Sun's birth cluster revisited. *Astron. Astrophys.* **670**, A105 (2023).

40. M. S. Fujii, S. Portegies Zwart, The formation and dynamical evolution of young star clusters. *Astrophys J.* **817**, 4 (2016).

41. M. S. Fujii, T. R. Saitoh, Y. Hirai, L. Wang, SIRIUS project. III. Star-by-star simulations of star cluster formation using a direct N-body integrator with stellar feedback. *Publ. Astron. Soc. Jpn.* **73**, 1074–1099 (2021).

42. S.-i. Inutsuka, T. Inoue, K. Iwasaki, T. Hosokawa, The formation and destruction of molecular clouds and galactic star formation. An origin for the cloud mass function and star formation efficiency. *Astron. Astrophys.* **580**, A49 (2015).

43. D. Arzoumanian, S. Arakawa, M. I. N. Kobayashi, K. Iwasaki, K. Fukuda, S. Mori, Y. Hirai, M. Kunitomo, M. S. N. Kumar, E. Kokubo, Insights on the Sun birth environment in the context of star cluster formation in hub-filament systems. *Astrophys J.* **947**, L29 (2023).

44. F. Palla, S. W. Stahler, Accelerating star formation in clusters and associations. *Astrophys J.* **540**, 255–270 (2000).

45. H. Störzer, D. Hollenbach, Photodissociation region models of photoevaporating circum-stellar disks and application to the proplyds in orion. *Astrophys J.* **515**, 669–684 (1999).

46. D. Dukes, M. R. Krumholz, Was the sun born in a massive cluster? *Astrophys J.* **754**, 56 (2012).

47. M. Reiter, Observational constraints on the likelihood of ^{26}Al in planet-forming environments. *Astron. Astrophys.* **644**, L1 (2020).

48. M. R. Krumholz, C. F. McKee, J. Bland-Hawthorn, Star clusters across cosmic time. *Annu. Rev. Astron. Astrophys.* **57**, 227–303 (2019).

49. B. S. Gaudi, S. Seager, B. Mennesson, A. Kiessling, K. Warfield, K. Cahoy, J. T. Clarke, S. Domagal-Goldman, L. Feinberg, O. Guyon, J. Kasdin, D. Mawet, P. Plavchan, T. Robinson, L. Rogers, P. Scowen, R. Somerville, K. Stapelfeldt, C. Stark, D. Stern, M. Turnbull, R. Amini, G. Kuan, S. Martin, R. Morgan, D. Redding, H. P. Stahl, R. Webb, O. Alvarez-Salazar, W. L. Arnold, M. Arya, B. Balasubramanian, M. Baysinger, R. Bell, C. Below, J. Benson, L. Blais, J. Booth, R. Bourgeois, C. Bradford, A. Brewer, T. Brooks, E. Cady, M. Caldwell, R. Calvet, S. Carr, D. Chan, V. Cormarkovic, K. Coste, C. Cox, R. Danner, J. Davis, L. Dewell, L. Dorsett, D. Dunn, M. East, M. Effinger, R. Eng, G. Freebury, J. Garcia, J. Gaskin, S. Greene, J. Hennessy, E. Hilgemann, B. Hood, W. Holota, S. Howe, P. Huang, T. Hull, R. Hunt, K. Hurd, S. Johnson, A. Kissil, B. Knight, D. Kolenz, O. Kraus, J. Krist, M. Li, D. Lisman, M. Mandic, J. Mann, L. Marchen, C. Marrese-Reading, J. McCready, J. McGown, J. Missun, A. Miyaguchi, B. Moore, B. Nemati, S. Nikzad, J. Nissen, M. Novicki, T. Perrine, C. Pineda, O. Polanco, D. Putnam, A. Qureshi, M. Richards, A. J. Eldorado Riggs, M. Rodgers, M. Rud, N. Saini, D. Scalisi, D. Scharf, K. Schulz, G. Serabyn, N. Sigrist, G. Sikkia, A. Singleton, S. Shaklan, S. Smith, B. Southerd, M. Stahl, J. Steeves, B. Sturges, C. Sullivan, H. Tang, N. Taras, J. Tesch, M. Therrell, H. Tseng, M.

Valente, D. Van Buren, J. Villalvazo, S. Warwick, D. Webb, T. Westerhoff, R. Wofford, G. Wu, J. Woo, M. Wood, J. Ziemer, G. Arney, J. Anderson, J. Maíz-Apellániz, J. Bartlett, R. Belikov, E. Bendek, B. Cenko, E. Douglas, S. Dulz, C. Evans, V. Faramaz, Y. K. Feng, H. Ferguson, K. Follette, S. Ford, M. García, M. Geha, D. Gelino, Y. Götberg, S. Hildebrandt, R. Hu, K. Jahnke, G. Kennedy, L. Kreidberg, A. Isella, E. Lopez, F. Marchis, L. Macri, M. Marley, W. Matzko, J. Mazoyer, S. McCandliss, T. Meshkat, C. Mordasini, P. Morris, E. Nielsen, P. Newman, E. Petigura, M. Postman, A. Reines, A. Roberge, I. Roederer, G. Ruane, E. Schwieterman, D. Sirbu, C. Spalding, H. Teplitz, J. Tumlinson, N. Turner, J. Werk, A. Wofford, M. Wyatt, A. Young, R. Zellem, The Habitable Exoplanet Observatory (HabEx) Mission Concept Study Final Report. arXiv:2001.06683 [astro-ph.IM] (2020).

50. X. Tang, R. A. Chevalier, Shock evolution in non-radiative supernova remnants. *Mon. Not. R. Astron. Soc.* **465**, 3793–3802 (2017).

51. S. Orlando, A. Wongwathanarat, H. T. Janka, M. Miceli, S. Nagataki, M. Ono, F. Bocchino, J. Vink, D. Milisavljevic, D. J. Patnaude, G. Peres, Evidence for past interaction with an asymmetric circumstellar shell in the young SNR Cassiopeia A. *Astron. Astrophys.* **666**, A2 (2022).

52. R. A. Chevalier, Young circumstellar disks near evolved massive stars and supernovae. *Astrophys J.* **538**, L151–L154 (2000).

53. N. Ouellette, S. J. Desch, J. J. Hester, Injection of supernova dust in nearby protoplanetary disks. *Astrophys J.* **711**, 597–612 (2010).

54. S. E. Woosley, T. A. Weaver, The evolution and explosion of massive stars. II. Explosive hydrodynamics and nucleosynthesis. *Astrophys. J. Suppl.* **101**, 181 (1995).

55. M. Matsuura, E. Dwek, M. Meixner, M. Otsuka, B. Babler, M. J. Barlow, J. Roman-Duval, C. Engelbracht, K. Sandstrom, M. Lakicevic, J. T. van Loon, G. Sonneborn, G. C. Clayton, K. S. Long, P. Lundqvist, T. Nozawa, K. D. Gordon, S. Hony, P. Panuzzo, K. Okumura, K. A. Misselt, E. Montiel, M. Sauvage, Herschel detects a massive dust reservoir in supernova 1987A. *Science.* **333** 1258–1261 (2011).

56. M. Shahbandeh, A. Sarangi, T. Temim, T. Szalai, O. D. Fox, S. Tinyanont, E. Dwek, L. Dessart, A. V. Filippenko, T. G. Brink, R. J. Foley, J. Jencson, J. Pierel, S. Zsíros, A. Rest, W. Zheng, J. Andrews, G. C. Clayton, K. De, M. Engesser, S. Gezari, S. Gomez, S. Gonzaga, J. Johansson, M. Kasliwal, R. Lau, I. De Looze, A. Marston, D. Milisavljevic, R. O'Steen, M. Siebert, M. Skrutskie, N. Smith, L. Strolger, S. D. Van Dyk, Q. Wang, B. Williams, R. Williams, L. Xiao, Y. Yang, JWST observations of dust reservoirs in type IIP supernovae 2004et and 2017eaw. *Mon. Not. R. Astron. Soc.* **523**, 6048–6060 (2023).

57. S. J. Smartt, Observational constraints on the progenitors of core-collapse supernovae: The case for missing high-mass stars. *Publ. Astron. Soc. Aust.* **32**, e016 (2015).

58. S. F. Portegies Zwart, S. L. W. McMillan, M. Gieles, Young massive star clusters. *Annu. Rev. Astron. Astrophys.* **48**, 431–493 (2010).

59. H. C. Plummer, On the problem of distribution in globular star clusters. *Mon. Not. R. Astron. Soc.* **71**, 460–470 (1911).

60. P. Kroupa, The initial mass function of stars: Evidence for uniformity in variable systems. *Science* **295**, 82–91 (2002).

61. T. Lee, F. H. Shu, H. Shang, A. E. Glassgold, K. E. Rehm, Protostellar cosmic rays and extinct radioactivities in meteorites. *Astrophys J.* **506**, 898–912 (1998).

62. Y. Fujimoto, M. R. Krumholz, S. Tachibana, Short-lived radioisotopes in meteorites from Galactic-scale correlated star formation. *Mon. Not. R. Astron. Soc.* **480**, 4025–4039 (2018).

63. D. F. Cioffi, C. F. McKee, E. Bertschinger, Dynamics of radiative supernova remnants. *Astrophys J.* **334**, 252 (1988).

64. S. W. Kinoshita, F. Nakamura, B. Wu, Star formation triggered by shocks. *Astrophys J.* **921**, 150 (2021).

65. S. Desch, N. Miret-Roig, The Sun's birth environment: Context for meteoritics. *Space Sci. Rev.* **220**, 76 (2024).

66. K. D. McKeegan, M. Chaussidon, F. Robert, Incorporation of short-lived ^{10}Be in a calciumaluminum-rich inclusion from the allende meteorite. *Science* **289**, 1334–1337 (2000).

67. E. T. Dunham, M. Wadhwa, S. J. Desch, M. C. Liu, K. Fukuda, N. Kita, A. T. Hertwig, R. L. Hervig, C. Defouilloy, S. B. Simon, J. Davidson, D. L. Schrader, Y. Fujimoto, Uniform initial ^{10}Be/^{9}Be inferred from refractory inclusions in CV3, CO3, CR2, and CH/CB chondrites. *Geochim. Cosmochim. Acta* **324**, 194–220 (2022).

68. B. A. L. Gaches, S. Walch, S. S. R. Offner, C. Münker, Aluminum-26 enrichment in the surface of protostellar disks due to protostellar cosmic rays. *Astrophys J.* **898**, 79 (2020).

69. F. H. Shu, H. Shang, T. Lee, Toward an astrophysical theory of chondrites. *Science* **271**, 1545–1552 (1996).

70. S. J. Desch, M. A. Morris, H. C. Connolly Jr., A. P. Boss, A critical examination of the X-wind model for chondrule and calcium-rich, aluminum-rich inclusion formation and radionuclide production. *Astrophys J.* **725**, 692–711 (2010).

71. S. Ekström, C. Georgy, P. Eggenberger, G. Meynet, N. Mowlavi, A. Wyttenbach, A. Granada, T. Decressin, R. Hirschi, U. Frischknecht, C. Charbonnel, A. Maeder, Grids of stellar models with rotation. I. Models from 0.8 to 120 $M_{\odot}$ at solar metallicity (Z = 0.014). *Astron. Astrophys.* **537**, A146 (2012).

72. A. P. Boss, S. I. Ipatov, S. A. Keiser, E. A. Myhill, H. A. T. Vanhala, Simultaneous triggered collapse of the presolar dense cloud core and injection of short-lived radioisotopes by a supernova shock wave. *Astrophys J.* **686**, L119–L122 (2008).

73. R. Fukai, S. Arakawa, Assessment of Cr isotopic heterogeneities of volatile-rich asteroids based on multiple planet formation models. *Astrophys J.* **908**, 64 (2021).
